# Supplementary material for: Atomic mechanism of near threshold fatigue crack growth in vacuum
Source: Nat Commun. 2022 Feb 10;13:812. doi: 10.1038/s41467-022-28481-8 (PMC8831527; doi:10.1038/s41467-022-28481-8)
Supplement: Supplementary file 1 — Supplementary Information [file 41467_2022_28481_MOESM1_ESM.pdf]

Supplementary Information for

Atomic mechanism of near threshold fatigue crack growth in vacuum

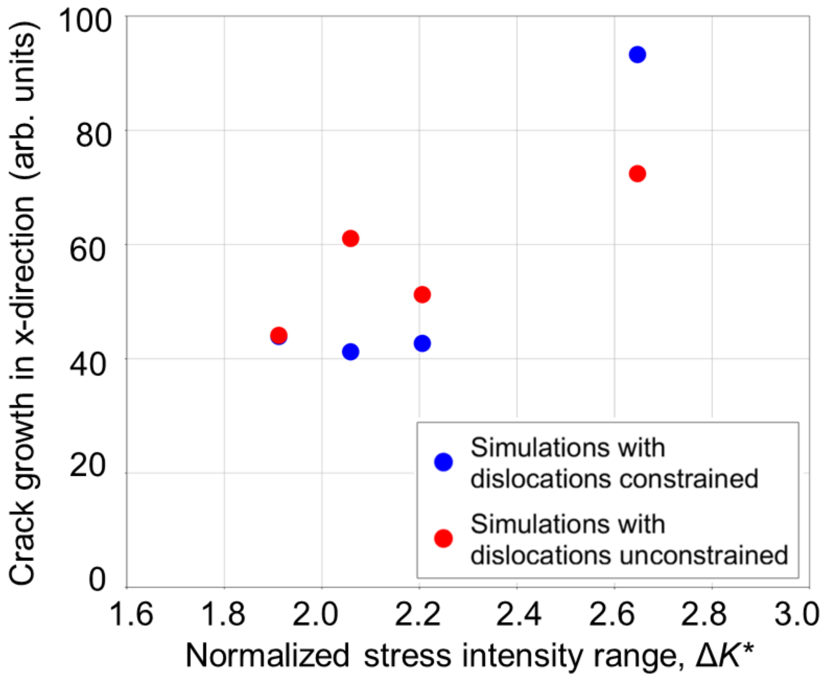

Supplementary Figure 1: Difference in crack growth at arrest between cases with dislocations constrained and unconstrained from passing between atomistic and continuum domains at various Δ*K values.** Passing dislocations from atomistic to continuum did not promote crack growth. Distances are normalized with respect to the magnitude of the Burgers vector, b, and stress intensity ranges are normalized by the stress intensity required to nucleate the first dislocation, $\Delta K^* = \Delta K_I / K_I^{nuc}$
